# Cervical neuroendocrine carcinoma in the third trimester: a rare case report and literature review

**DOI:** 10.1186/s12884-023-05900-2

**Published:** 2023-08-10

**Authors:** Gezi Chen, Kai Huang, Jinglei Sun, Lei Yang

**Affiliations:** 1 Department of Obstetrics, The First Affiliated Hospital of Zhengzhou University, Zhengzhou, China; 2 Center for Reproductive Medicine, The First Affiliated Hospital of Zhengzhou University, Zhengzhou, China; 3 Henan Key Laboratory of Reproduction and Genetics, The First Affiliated Hospital of Zhengzhou University, Zhengzhou, China; 4 Henan Provincial Obstetrical and Gynecological Diseases (Reproductive Medicine) Clinical Research Center, The First Affiliated Hospital of Zhengzhou University, Zhengzhou, China

**Keywords:** Cervical neuroendocrine carcinoma (CNECC), Gynecological tumor screening, Pregancy, Prognosis, Treatment strategies

## Abstract

**Background:**

The incidence of cervical neuroendocrine carcinoma (CNECC) during pregnancy is extremely rare. Insufficient awareness of gynecological tumor screening, as well as atypical and insidious initial clinical symptoms, may result in diagnostic delays and misdiagnosis. There is no standardized treatment for cervical cancer in pregnancy. Herein, we present a case of cervical neuroendocrine carcinoma diagnosed in the third trimester of pregnancy.

**Case presentation:**

A 26-year-old female at 30 1/7 weeks of gestation presented with lower back and sacroiliac joint pain, abdominal distension, and lower limb dyskinesia. A pelvic examination revealed a large fungating gray mass that encompassed the entire cervix, with cervical contact bleeding testing positive. Imaging studies showed a significant cervical mass, diffuse liver changes, and metastasis to multiple sites. Biopsy results revealed poorly differentiated cervical carcinoma exhibiting high-grade neuroendocrine features, consistent with a diagnosis of large cell neuroendocrine carcinoma. The patient was diagnosed with stage IVB CNECC with HPV18 (+), and due to the gestational age of the fetus and her deteriorating condition, she underwent cesarean section delivery after receiving fetal lung maturation therapy. Following surgery, eight cycles of neoadjuvant chemotherapy were applied. Unfortunately, she succumbed to multiple tumor metastases six months post-treatment. Despite this tragic outcome, the infant was discharged in a healthy condition.

**Conclusions:**

CNECC during pregnancy, particularly the large-cell type, is an ultra-rare condition with poor prognosis. This case highlights the importance of individualized treatment approach and the need for better screening, early detection, and treatment. Given the rarity of the disease, further research is warranted to determine the prognostic factors and develop effective treatment strategies for this ultra-rare and aggressive malignancy.

## Background

Pregnancy complicated with cervical cancer represents a commonly occurring gynecological malignancy tumor. While cervical neuroendocrine carcinoma is a relatively rare pathologic type of cervical cancer, it is histologically characterized by small cell type, large cell type, typical carcinoid, and atypical carcinoid. Among these subtypes, small-cell neuroendocrine carcinoma of the cervix (SCNCC) is the predominant type. The incidence of cervical neuroendocrine carcinoma (CNECC) during pregnancy, particularly the large-cell type, is an ultra-rare condition. It has been reported that CNECC is linked to Human Papilloma Virus (HPV) infection, particularly with HPV 18 [1]. Patients with CNECC are prone to early invasion and metastasis, with the pelvic cavity, bone, lung, brain, and liver being the most common sites of late-stage metastases [2]. Given the rarity of this disease, most cases have been reported on a small scale and from a single institution. There is currently limited knowledge regarding prognostic factors such as the stage of disease (tumor size), lymph node involvement, duration of pregnancy, and histological subtypes. Unfortunately, diagnosis may already occur in the terminal stages of the disease, resulting in an extremely poor prognosis. The prognosis of CNECC is significantly worse than that of cervical squamous cell carcinoma of the same stage, with the 5-year overall survival rate being approximately 30% [3].

## Case presentation

A 26-year-old female, gravida-3 para-1, at 30 1/7 weeks of gestation presented to the Emergency Department with symptoms of lower back and sacroiliac joint pain, along with abdominal distension. Her medical history revealed that she had been admitted to a county hospital during the second month of her pregnancy due to hyperemesis gravidarum. After one week of supportive therapy, her symptoms mostly subsided. During the fourth month of her pregnancy, fetal movement was observed, and she had regular antenatal care with normal NT (Nuchal Translucency), low-risk Down’s screening, and normal OGTT (Oral Glucose Tolerance Test) results. Four-dimensional ultrasonography suggested an intrauterine pregnancy with a single alive fetus in the breech position and strong light spots in the left ventricle of the fetal heart. Approximately four weeks before admission to our hospital, she experienced progressively worsening low back pain, for which a local doctor recommended observation and rest considering her enlarged pregnancy abdomen.

The patient presented with abdominal distension, lower limb dyskinesia, and severe ache, which appeared one day before admission. A pelvic examination conducted in the county hospital revealed the presence of a large fungating gray mass that encompassed the entire cervix, with cervical contact bleeding testing positive. The patient exhibited symptoms such as a pale face, poor spirit and appetite, normal sleep, dry stool occurring once every three days, normal urination, and a weight loss of 10 kg compared to before her pregnancy, since the onset of the disease.

**Fig. 1:**
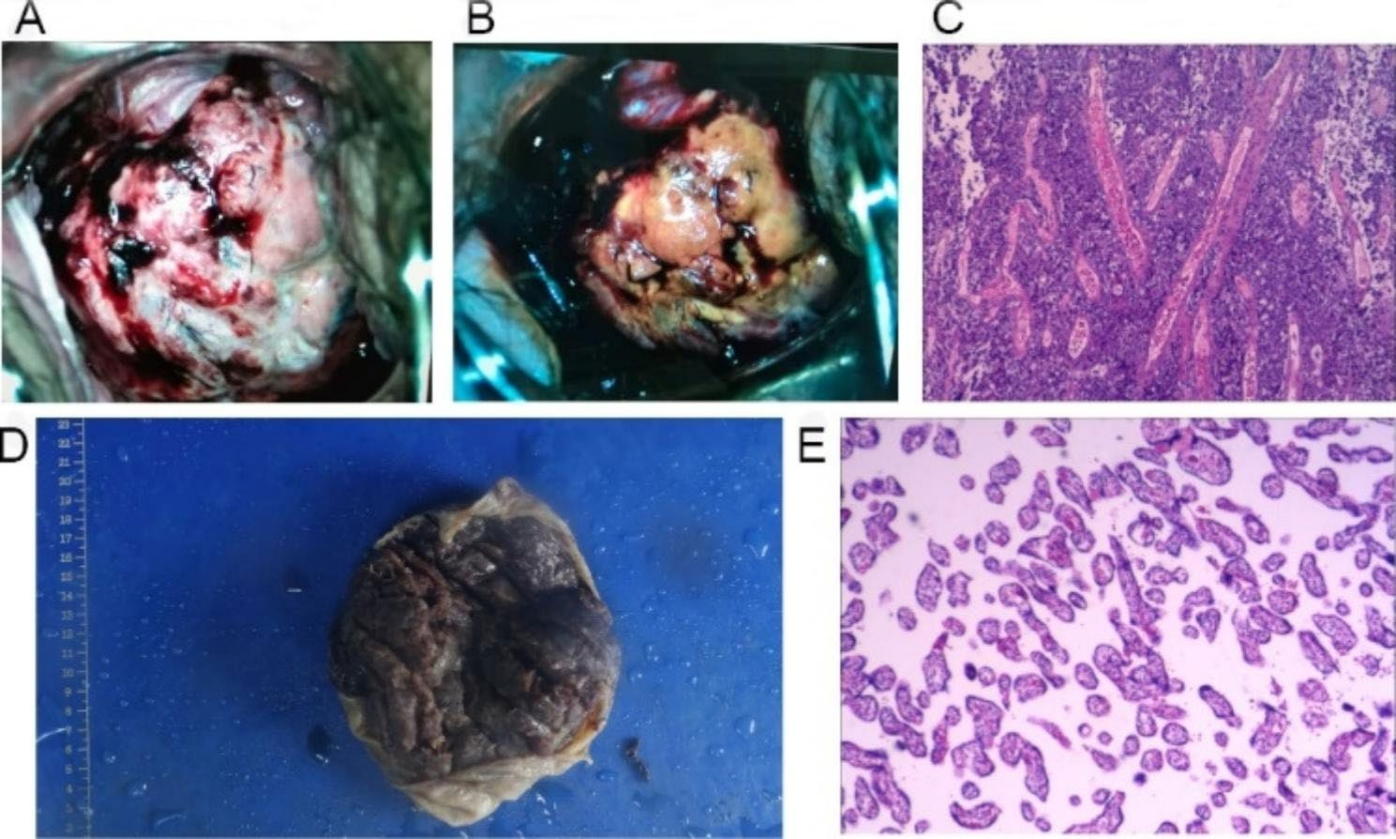
Cervical neuroendocrine carcinoma in pregnancy. (**A**) Acetowhite test of the cervix. (**B**) The coloring test of the cervix. (**C**) HE staining of Cervical biopsy tissues. (**D**) Maternal placenta. (**E**) HE staining of Placenta

The patient received multiple blood transfusions due to severe anemia. Ultrasound examination revealed the presence of a diffuse echo-change approximately 13.6 cm by 11.5 cm in size in the liver, which appeared to be a solid-cystic mass that could potentially be a metastatic tumor. To confirm the diagnosis, cervical cancer screening and biopsy were conducted (Fig. 1A-C). Immunohistochemical analysis was performed and the results revealed positive expression of AE1/AE3, CK8/18, CAM5.2, P16, PAX-8, Syn, CD56, and CgA. Additionally, the tumor showed high proliferation index with over 90% of cells staining positively for Ki-67.

**Fig. 2:**
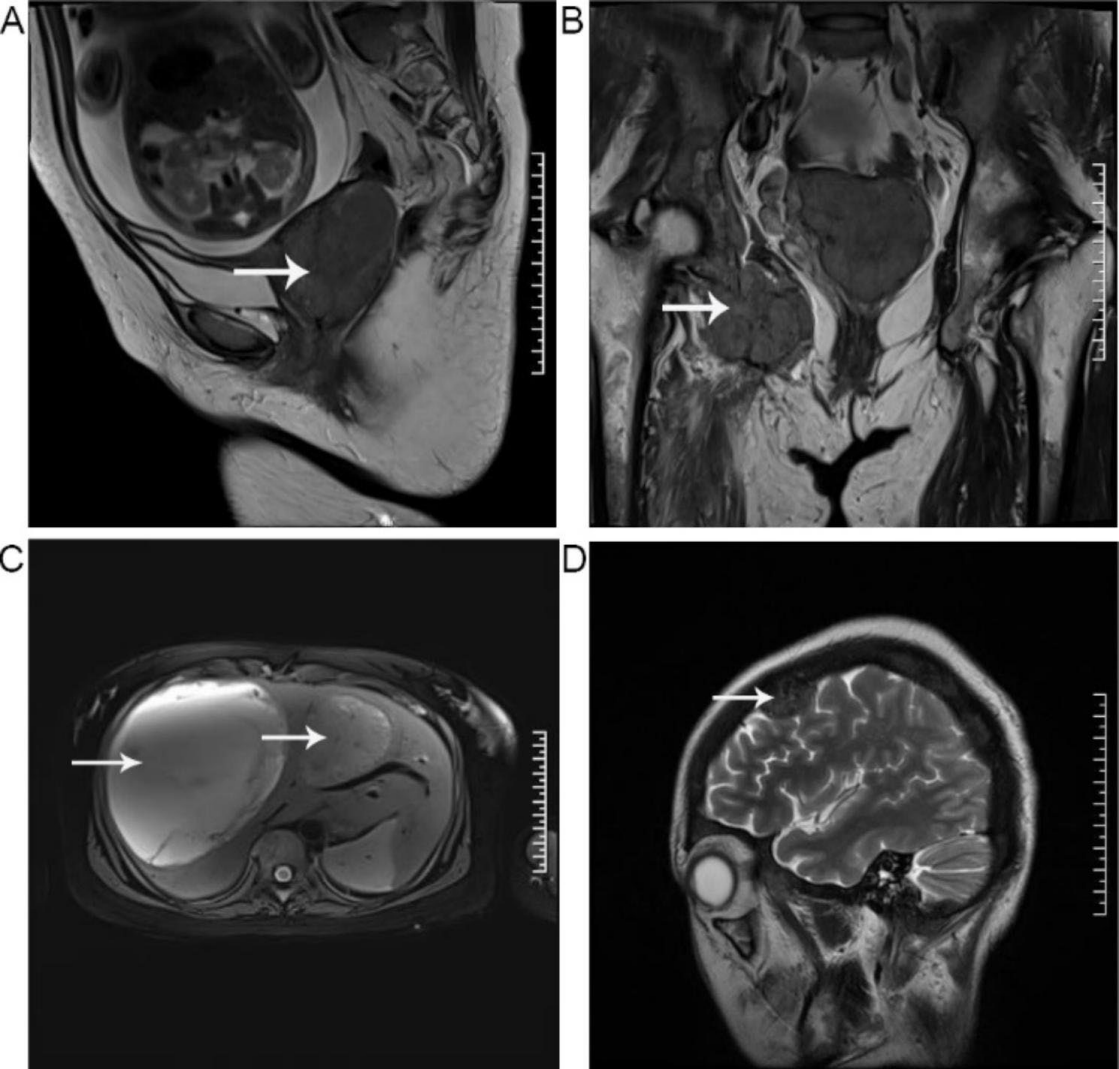
Magnetic Resonance Imaging of tumor and metastases. (**A**) Cervix. (**B**) Ilium and ischium. (**C**) Liver. (**D**) Brain

The magnetic resonance imaging (MRI) results indicated the presence of a significant cervical mass measuring 5.3 × 6.6 × 7.2 cm. In addition, abnormal signals were detected in multiple other locations, including the right scapula, clavicle, multiple ribs, thoracic vertebrae, lung, lumbar vertebrae, right femoral lesser trochanter, bilateral iliac crest, right ischium, acetabulum, and surrounding soft tissue. Multiple cystic solid masses were also detected in the liver, with the largest mass measuring approximately 13.5 × 13.7 × 14.8 cm, which may suggest metastasis (Fig. 2).

A multidisciplinary team consisting of specialists in gynecology, oncology, maternal-fetal medicine, neonatology, interventional medicine, palliative care, and pain management was convened to discuss the case. It was unanimously agreed that a biopsy was warranted, which subsequently revealed poorly differentiated cervical carcinoma exhibiting high-grade neuroendocrine features that were consistent with a diagnosis of large cell neuroendocrine carcinoma. The collective diagnostic results confirmed that the patient was suffering from a rare form of cervical cancer, specifically cervical neuroendocrine carcinoma (CNECC) with HPV18(+) and stage IVB. Considering the gestational age of the fetus and the patient’s deteriorating pain and desire to terminate the pregnancy, she declined neo-adjuvant chemotherapy (NACT) and elected to undergo cesarean section delivery at 31 4/7 weeks gestation after receiving fetal lung maturation therapy. The placenta was unremarkable and no tumors were found (Fig. 1D-E). The neonate had Apgar scores of 10 and 10 at 1 and 5 min, respectively, weighed 1700 g, and was subsequently transferred to the neonatal intensive care unit under the care of a neonatologist.

Following surgery, the patient was treated with three cycles of carboplatin and etoposide chemotherapy, followed by an additional five cycles of paclitaxel, nedaplatin, and bevacizumab chemotherapy, with each course administered at 3-week intervals. Unfortunately, she succumbed to multiple tumor metastases six months post-treatment, leading to cardiopulmonary failure, infection, and cachexia. Despite this tragic outcome, the infant was discharged in a healthy condition.

## Discussion and conclusions

Cervical cancer is a relatively infrequent yet significant concern during pregnancy, with a reported incidence ranging from 0.05 to 0.1% [4]. Squamous cell carcinoma constitutes a majority (75–80%) of cervical tumors, while adenocarcinomas account for the remaining 20–25%. A rare subtype of mucinous adenocarcinoma of the cervix, not associated with Human Papilloma Virus, arises from the mucin-producing columnar epithelium of the endocervical glands and accounts for 3% of cervical adenocarcinomas [5]. During pregnancy, cervical neuroendocrine carcinoma (CNECC) is an exceptionally rare occurrence with highly malignant properties and a propensity for rapid progression and lymph node metastasis [6–8].

Due to the absence of routine gynecological examination in early pregnancy, misdiagnosis of cervical cancer may occur. To screen for cervical cancer, HPV genotyping should be performed as the first step, as recommended for non-pregnant patients [9]. If the patient tests positive for HPV 16 or 18, colposcopy is recommended. Conversely, if the patient tests negative for HPV 16 and 18 but positive for another high-risk HPV genotype, cervical exfoliated cytology should be performed. If the cytology reveals any epithelial abnormality greater than atypical squamous cells of undetermined significance (ASCUS), colposcopy is required. In cases where cytology is negative, annual follow-up is suggested. However, if the HPV test is negative, the recommended follow-up time is every 3 years [9]. In summary, HPV genotyping and cervical exfoliated cytology are the primary early screening methods for cervical cancer and are recommended for pre-pregnancy examination or the first prenatal examination. If necessary, a colposcopy biopsy is the preferred diagnostic test due to its accuracy. MRI can be utilized to evaluate tumor invasion and lymph node metastasis without increasing the risk of congenital malformations in the fetus [10].

This case report details the atypical presentation of cervical cancer in a young patient, who did not present with characteristic clinical manifestations such as vaginal bleeding or discharge. Rather, her first symptom was low back pain, which raised the possibility of bone metastasis. The patient initially sought medical attention at a local clinic where observation and rest were recommended due to an enlarged pregnancy abdomen. However, one month later, the patient’s symptoms had worsened and she presented to our hospital with rib pain and abdominal distension. Cervical biopsy confirmed the diagnosis of cervical cancer, but unfortunately, the disease had progressed to an advanced stage, precluding surgical intervention.

While the patient and her family expressed a desire to continue the pregnancy, conservative treatment for her condition was unsuccessful, with her pain continuing to worsen after 10 days. In response to the patient’s request, a cesarean section was performed. The postoperative management of the patient was not significantly different from that of non-gestational cervical cancer patients.

The management of cervical cancer in pregnant women remains controversial. A multidisciplinary approach is recommended to provide personalized treatment plans, considering various factors, including the stage of the disease, lymph node involvement, histological subtype, duration of pregnancy, and the patient’s preference regarding continuation of pregnancy and fetal outcomes. In particular, cervical neuroendocrine adenocarcinoma in pregnant women is associated with even worse outcomes.

For patients with stage IA1 cervical cancer who are less than 20 weeks pregnant, expectant treatment with regular cytology or colposcopy every 12 weeks is recommended, as the effect of pregnancy on tumor biology is still uncertain [11]. From 22 weeks of gestation onwards, several options are available, such as regular monitoring until delivery, delayed treatment after childbirth, or administering 2 to 3 courses of neo-adjuvant chemotherapy to control tumor progression until the fetus is mature. Pelvic lymph node dissection and neo-adjuvant chemotherapy can be considered for patients with stage IB2 cervical cancer and gestation less than 22 weeks [12]. The use of sentinel lymph node (SLN) biopsy in early cervical cancer has gained traction among non-pregnant women, with the 2020 NCCN guidelines suggesting its feasibility in patients with stage IAl and IA2 cervical cancer [13]. SLN imaging biopsy with ICG shows great potential but is not yet used extensively in pregnant patients with cervical cancer. Although infants born to mothers who have received neo-adjuvant chemotherapy are believed to be healthy, further research is necessary to confirm this finding.

In cases where pregnancy has progressed beyond 22 weeks, NACT remains the only option for pregnancy maintenance in patients with stage IB3 or above cervical cancer. However, the administration of NACT after 33 weeks of gestation is not advisable due to the high risk of preterm delivery [14]. Current literature and case reports demonstrate that NACT can significantly reduce and regress the cancer foci in some patients with locally advanced cervical cancer [15]. Delays in treatment should be avoided if cervical cancer is diagnosed in the first or second trimester, while treatment can be briefly postponed in the third trimester. Pregnancy termination can be considered at a selected time after fetal lung maturation, concurrent radiotherapy and chemotherapy can be utilized in the postpartum period, as in this case. Radiation therapy is not routinely recommended during pregnancy due to potential risks including spontaneous abortion, congenital malformations, and pediatric malignancies.

In the management of cervical cancer during pregnancy, the use of neo-adjuvant chemotherapy may lead to an increased risk of fetal malformation, which is related to the timing and number of NACT drugs administered. Previous studies have demonstrated comparable efficacy and adverse reactions between arterial and venous NACT administration [16]. However, current evidence on the fetal and neonatal outcomes of NACT remains limited by small sample sizes and short-term follow-up, highlighting the need for close medical monitoring throughout pregnancy and delivery. Further studies are necessary to establish the safety and efficacy of NACT in this context.

Numerous evidence-based guidelines have been established to manage cervical cancer in pregnancy, but for advanced cervical cancer, the management approach relies mostly on theory and expert opinion, as there is a dearth of large-scale, prospective, and randomized controlled trials.

Once diagnosed as cervical cancer in terminal pregnancy, cesarean section is the recommended method of delivery due to the risks associated with vaginal delivery, including tumor obstruction and recurrence of episiotomy site. Vaginal delivery has been associated with higher recurrence rates and lower overall survival rates compared to cesarean Section [17]. Corticosteroid administration is recommended for fetal lung maturation after 32 weeks of gestation. During cesarean section, a vertical uterine incision is preferred to maintain the integrity of the lower uterine segment and facilitate postoperative pathological staging. The presence of placental metastases should be carefully evaluated during the operation. The optimal timing of perinatal radical hysterectomy remains a subject of debate. In this case, a cesarean section was performed without additional surgery.

In the context of pregnancy, cervical neuroendocrine carcinoma is a rare and mostly fatal disease. However, no standardized treatment protocol has been established for this condition. To avoid misdiagnosis, clinicians should emphasize the importance of cervical cancer screening to pregnant women. Upon diagnosis, individualized treatment plans should be formulated based on the specific conditions of each patient. Given the ethical considerations and safety concerns for both the mother and the fetus, it is imperative to engage in sufficient communication with patients and their families. Improved screening, early detection, and treatment may enhance the survival rate of pregnant women with cervical neuroendocrine carcinoma. Consequently, additional clinical experience is required to establish a reliable clinical foundation and a definitive treatment strategy for this disease.

## Data Availability

The datasets used during the current study are available from the corresponding author on reasonable request.

## Abbreviations

CNECC: Cervical neuroendocrine carcinoma
SCNCC: Small-cell neuroendocrine carcinoma of the cervix
NT: Nuchal Translucency
OGTT: Oral Glucose Tolerance Test
HPV: Human Papilloma Virus
NACT: Neo-adjuvant chemotherapy ()
ASCUS: Atypical squamous cells of undetermined significance
SLN: Sentinel lymph node
